# 

**Published:** 1883-08

**Authors:** 


					﻿AUGUST, 1883.
THIRTIETH YEAR.
HALT’S
JOURNAL
OF
HEALTH
Guaranteed Circulation 200,000 Copies in 12* Months.
E. H. GIBBS, A.M., MB)., Editor.,
No. 21 CLINTON PLACE, EIGHTH STEEET NEW YORK.
TERMS: $1 a Year.
SiDile number, 10 cts.
				

